# Misidentification of *Diphyllobothrium* Species Related to Global Fish Trade, Europe

**DOI:** 10.3201/eid2011.140996

**Published:** 2014-11

**Authors:** Roman Kuchta, José-Guillermo Esteban, Jan Brabec, Tomáš Scholz

**Affiliations:** Institute of Parasitology, Biology Centre of the Academy of Sciences of the Czech Republic, České Budějovice, Czech Republic (R. Kuchta, J. Brabec, T. Scholz);; Facultad de Farmacia, Universidad de Valencia, Valencia, Spain (J.-G.Esteban)

**Keywords:** Cestoda, diphyllobothriosis, fish-borne disease, zoonoses, parasites, foodborne, Europe, Spain, South America

**To the Editor:** Diphyllobothriosis, infection by tapeworms of the genus *Diphyllobothrium* (Cestoda: Diphyllobothriidea) (*1*), is a well-known disease of humans. In Europe, infections caused by 3 species of *Diphyllobothrium* have recently been reported in humans: *D. latum* is considered to be the principal species infecting persons in Europe (*1*); 4 cases of *D. dendriticum* infection and 6 cases of *D. nihonkaiense* infection have also been reported (*2*,*3*). Except for those caused by *D. latum*, which is autochthonous in northeastern Europe and subalpine lakes, most of the cases in Europe have been imported or caused by consumption of fish imported from areas to which the parasites are endemic (*1*,*3*,*4*).

Diphyllobothriosis is not endemic to Spain, but 7 cases of *D. latum* infection have been reported there (Technical Appendix Table). Most recently, Pastor-Valle et al. confirmed, using molecular tools, an imported case of infestation by *Diplogonoporus balaenopterae* and 3 imported cases of diphyllobothriosis caused by *D. pacificum* (*5*), a tapeworm endemic to the Pacific coast of South America (*1*,*4*).

Specific identification of most human-infecting *Diphyllobothrium* tapeworms based on clinical material is virtually impossible (*1*,*3*); the only exception is identifying the Pacific broad tapeworm, *D. pacificum*. This tapeworm can be easily distinguished from other human-infecting diphyllobothriideans by the presence of pits alongside the median line on the ventral surface of its proglottids; smaller, more spherical, eggs; and the almost equatorial position of the genital pore, a feature that is markedly pre-equatorial in other species (Technical Appendix Figure 1). Several hundred cases of infection by this species have been reported from Peru, and a few reports have been made from Ecuador, Chile, and Japan (*1*). The life cycle of *D. pacificum* is not completely known, but several species of marine fish have been identified as sources of human infection in Peru (*4*).

We critically examined all recent records of diphyllobothriosis in Spain to clarify species identification because published morphologic data indicated misdiagnosis (Technical Appendix Table). Tapeworms detected in 2 recent human cases reported by Colomina et al. (*6*) and Esteban et al. (*7*), described as *D. latum*, resembled those of *D. pacificum* because of the morphology of proglottids and eggs (*6*,*7*). Therefore, we requested material of these cestodes for scrutiny. Morphologic and molecular evaluation (partial lsrDNA and *cox1* gene sequences; multiplex PCR testing by Wicht et al. (*8*), (Figure, Technical Appendix Figures 1, 2) actually confirmed that *D. pacificum* was misidentified as *D. latum* in both cases, despite the molecular identification through multiplex PCR.

**Figure:**
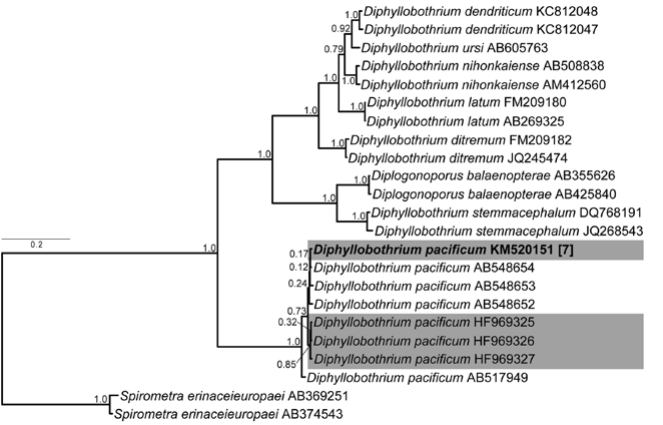
Bayesian inference phylogenetic analysis of selected human diphyllobothrideans based on cox1 gene analyzed as 3 independent data parts according to the nucleotide coding positions by using (GTR+G)(HKY)(GTR+G) evolutionary model setup in MrBayes (mrbayes.sourceforge.net). Topologies sampled every 1,000th generation over 4 runs and 20,000,000 generations, burn-in 25%. *Diphyllobothrium pacificum* identified in Spain marked in gray; new sequence is in bold type. Scale bar indicates nucleotide substitutions per site.

No voucher specimens for re-identification were available for another 2 alleged cases of *D. latum* infection (Technical Appendix Table). However, the eggs reported in the study by Gil-Setas et al. were more similar in shape and size to those of *D. nihonkaiense* or *Diplogonoporus balaenoterae* than to those of *D. latum* (*9*).

*D. latum* is the principal causative agent of human diphyllobothriosis; its fish intermediate hosts are perch, pike, burbot, and ruff in Europe (*1*,*4*). Other fish, such as salmonids and marine fish, cannot transmit this parasite and serve as intermediate hosts of other species of *Diphyllobothrium* and *Diplogonoporus* (*4*).

The information on the spectrum of its fish intermediate hosts of *D. pacificum* is limited. From very scarce anamnestic data about individual case-patients infected with *D. pacificum* in Spain, it is not possible to unravel the actual source of their infection. However, it is obvious that the recent emergence of diphyllobothriosis caused by nonendemic species such as *D. pacificum*, *D. dendriticum* (*3*), *D. nihonkaiense* (*2*), and *D. balanopterae* (*5*) is related to the global importation of fish that have not been frozen. If the fish are merely chilled, plerocercoids of diphyllobothriids may survive for several days (*10*).

Spain is the third largest importer of fish and seafood in the world; the value of fish products imported from >104 countries reached $7 billion (US) in 2011 and increases continuously. More than 200,000 tons of fresh or chilled fish, which may serve as source of human fishborne diseases if eaten raw or undercooked, are imported to Spain every year. The fourth largest importer is Ecuador, the sixth is Chile, and the seventh is Peru; *D. pacificum* is endemic to each of these countries (*4*).

In the present study, we confirmed human infections with the Pacific broad tapeworm, *D. pacificum*, in Europe, but it is highly probable that this species can be introduced anywhere through the importation of fresh or chilled fish from the Pacific coast of South America. This has implications for food safety rules and human health risk measures taken by national health and veterinary agencies. Regarding adequate processing of clinical samples and their preservation for morphologic and genetic evaluation, we strongly recommend fixation of positive fecal samples with eggs or segments (proglottids) immediately with 96%–99% molecular grade (i.e., not denatured) ethanol for future molecular diagnosis (*1*,*4*,*8*).

Technical Appendix A summary of Diphyllobothrium and Diplogonoporus cases in Spain, photomicrographs of features of diphyllobothriids, and results of testing for human-infecting Diphyllobothrium spp.
