# Supplementary material for: Misidentification of Diphyllobothrium Species Related to Global Fish Trade, Europe
Source: Emerg Infect Dis. 2014 Nov;20(11):1955–7. doi: 10.3201/eid2011.140996 (PMC4214320; doi:10.3201/eid2011.140996)
Supplement: Technical Appendix — A summary of Diphyllobothrium and Diplogonoporus cases in Spain, photomicrographs of features of diphyllobothriids, and results of testing for human-infecting Diphyllobothrium spp. [file 14-0996-Techapp-s1.pdf]

DOI: http://dx.doi.org/10.3201/eid2011.140996

Misidentification of *Diphylobothrium* Species Related to Global Fish Trade, Europe

Technical Appendix

Technical Appendix Table. Survey of cases of *Diphyllobothrium diphyllobothriosis* and *diplogonoporias* infections, Spain, 1983–2014*

Tapeworm species, subspecies	No. cases	Study	Remarks
D. latum†	1	Zerolo et al. 1983 (1)	No data or material available‡
Diplogonoporus balanopterae	1	Clavel et al. 1997 (2)	Indicated by proglottid morphology
D. nihonkaiense or *Diplogonoporus balanopterae*†	1	Gil-Setas et al. 2004 (3)	Indicated by egg morphology; no material available
D. pacificum	1	Colomina et al. 2002 (*4*)	Re-identified as '*D. latum*' by morphology
D. pacificum	1	Esteban et al. 2013 (*5*)	Re-identified as '*D. latum*' by DNA
D. pacificum	3	Pastor-Valle et al. 2014 (6)	DNA identification‡
Diplogonoporus balanopterae	1	Pastor-Valle et al. 2014 (*6*)	DNA identification‡

* *D.*, *Diphyllobothrium.*
†Confirmation of identity not possible.
‡No morphological data provided.

References

1. Zerolo FJ, Ibarra A, Escauriaza J, Ruiz P, Costan G. *Diphyllobothrium latum*: consideraciones a propósito de un caso en Córdoba. Espagnol. 1983;2:76–8.

2. Clavel A, Bargues MD, Castillo FJ, Rubio MC, Mas-Coma S. Diplogonoporiasis presumably introduced into Spain: first confirmed case of human infection acquired outside the Far East. Am J Trop Med Hyg. 1997;57:317–20. PubMed

3. Gil-Setas A, Mazón A, Pascual P, Sagua H. Helminthiasis in a 71-year-old man, an infrequent condition in our setting [in Spanish]. Enferm Infecc Microbiol Clin. 2004;22:553–4. http://dx.doi.org/10.1157/13067624

4. Colomina J, Villar J, Esteban G. Asymptomatic infection by *Diphyllobothrium latum* in a Spanish 3-year-old child [in Spanish]. Med Clin (Barc). 2002;118:279. PubMed http://dx.doi.org/10.1016/S0025-7753(02)72359-2

5. Esteban JG, Munoz-Antoli C, Borras M, Colomina J, Toledo R. Human infection by a "fish tapeworm", *Diphyllobothrium latum*, in a non-endemic country. Infection. 2014;42:191–4. PubMed http://dx.doi.org/10.1007/s15010-013-0491-2</jrn>

6. Pastor-Valle J, González LM, Martín-Clemente JP, Merino FJ, Gottstein B, Gárate T. Molecular diagnosis of diphyllobothriasis in Spain, most presumably acquired via imported fish, or sojourn abroad. New Microbes and New Infections. 2014;2:1–6. http://dx.doi.org/10.1002/2052-2975.28

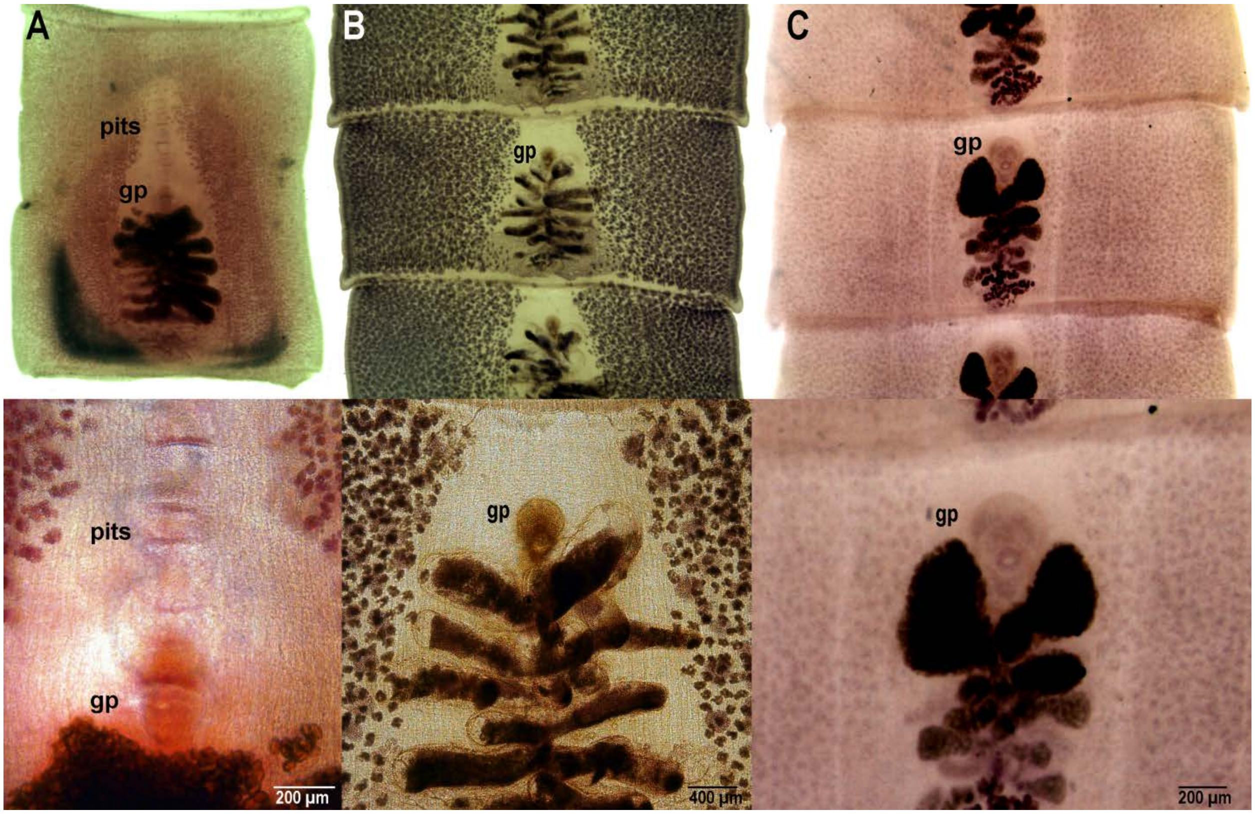

Technical Appendix Figure 1. Photomicrographs of segments of diphyllobothriids from human. A) *Diphyllobothrium pacificum* reported by Esteban et al. (2013); B) *D. latum* (experimental human infection); C) *D. nihonkaiense* (case from Japan). gp, genital pore; pits, longitudinal pits anterior to the genital atrium typical of D. pacificum.

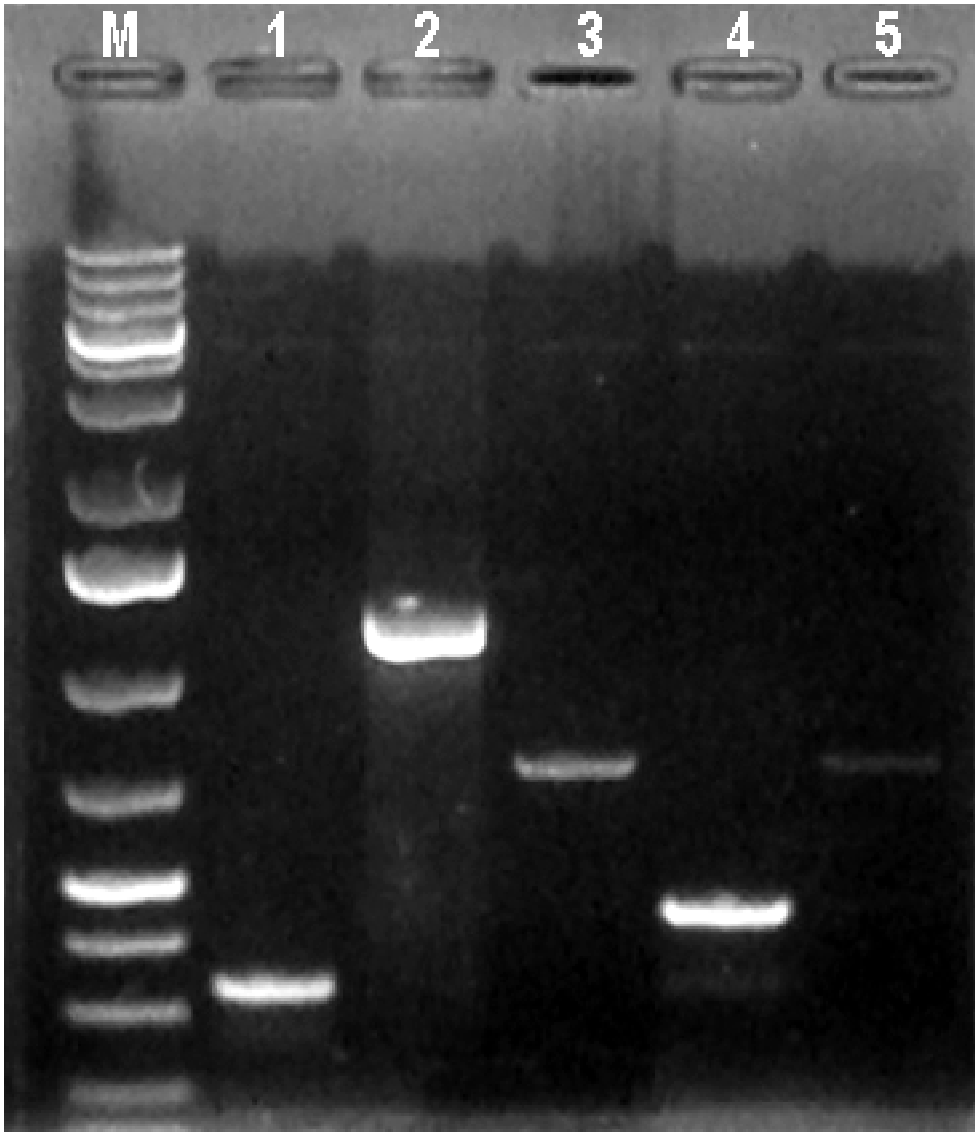

Technical Appendix Figure 2**.** Differential diagnosis of human-infecting *Diphyllobothrium* spp. by multiplex PCR after Wicht et al. 2010. Lane M, GeneRuler 1kb Plus Ladder (ThermoScientific); lane 1, *D. dendriticum* positive control (318 bp); lane 2, *D. nihonkaiense* positive control (1232 bp); lane 3, *D. pacificum* positive control (727 bp); lane 4, *D. latum* positive control (437 bp); lane 5; Esteban et al. (2013) amlicon.
